# Adaptive immunity to SARS-CoV-2

**DOI:** 10.1093/oxfimm/iqaa003

**Published:** 2020-07-09

**Authors:** Daniel M Altmann

**Affiliations:** Department of Immunology and Inflammation, Faculty of Medicine, Imperial College, Hammersmith Hospital, London W12 0NN, UK

**Keywords:** COVID-19, SARS-CoV-2, coronavirus, T cell, antibody, cytokines, lung, immunopathology, vaccine, correlate of protection

## Abstract

The majority of severe acute respiratory syndrome coronavirus 2 (SARS-CoV-2 exposed individuals mount an antibody response within around 2-weeks and spike antigen-binding responses correlate well with functional virus neutralization. A minority makes little detectable antibody, generally those with either very mild/asymptomatic disease or those with severe/lethal infection. However, in general, antibody titre correlates with viral load and duration of exposure. There is evidence for cross-reactivity with the other human coronaviruses, though the functional impact of this is as yet unclear. Therapeutic use of neutralizing monoclonal antibodies offers potential for clinical use. While there is evidence for neutralizing antibody as a correlate of protection, some cases indicate the potential for full recovery in the absence of antibody. Studies of T-cell immunity following acute infection show CD4 and CD8 responses to epitopes across diverse viral antigens, possible cross-reactivity with epitopes from the common cold human coronaviruses and large-scale activation. However, in severe cases, there is evidence for T-cell lymphopaenia as well as expression of exhaustion markers. Analysis of serum biomarkers of disease severity implicates a hyperinflammatory contribution to pathogenesis, though this has not been mechanistically delineated beyond a likely role of raised IL-6, considered a therapeutic target. Despite rapid progress, there remain pressing unknowns. It seems likely that immune memory to SARS-CoV-2 may be relatively short lived, but this will need longitudinal investigation. Also, this is a disease of highly variable presentation and time course, with some progressing to protracted, chronic symptoms, which are not understood. The contribution of immunopathological mechanisms to tissue damage, whether in the lung, kidney, heart or blood vessels, is unclear. The immunology underlying the differential susceptibility between the very young and the very old is unresolved, a question with ramifications for vaccine roll-out. The greatest challenge relates to rapid generation, testing and manufacture of vaccines that are immunogenic, protective (at least from symptomatic disease) and safe—a challenge that looks achievable.

Since the end of 2019, cases of COVID-19, the disease caused by SARS-CoV-2 viral infection, have escalated to a global pandemic. Sequencing and characterization of the virus have facilitated considerable advances in knowledge of host immunity from a standing start, aided in no small part by clinical immunology studies of initial patient cohorts hospitalized with acute disease during early stages of the pandemic. Given public and governmental concern over the risks and future management of infection, immunology research has been placed in the spotlight, with intense curiosity and scrutiny about many specific aspects of the immune response to this viral infection: when does immunity develop, what are the correlates of protection, what is the temporal relationship between immunity and infectivity, do all develop protective immunity and can reinfection occur, what part is played by immunopathology in pathological damage to the lungs and other organs? On top of this has been impatience for updates on progress in rapid resolution of the translational challenges posed by global roll-out of reliable antibody serodiagnostics and of safe reliable vaccines [1–4]. Immunology has never had to grapple with questions of this enormity under such time pressure.

Among the countless manifestations of the ‘new normal’ has been an overturning of conventions for publishing so as to address the pressure for data updates in real time: the tendency has been for data to emerge as soon as it is generated, on social media, then within days or weeks posted on repositories such as BioRxiv as a non-peer-reviewed preprint, then subsequently snapped up for full publications in prestigious journals. There have been many consequences of this publishing revolution. There has been a vibrant, refreshing foreshortening of the publication timeline. This is a field that had developed norms whereby big papers necessitated the pooled work of perhaps a score of scientists over 5 years of funded research, submitting a manuscript for laboured, iterative, peer-review stretching over 6–12 months, so that the full cycle from concept, to funding, to research, to publication might be 7 years plus. In the ‘new normal’, some of the highest profile papers have used standard, pre-existing technologies such as multiparameter flow cytometry panels and RNAseq pipelines to describe and define immune parameters in patients hospitalized in January and February, the papers reporting them appearing in March and April. While there may indeed be a price for reduced rigour in peer review, many might argue that the scrutiny of a scientific peer group via social media has gone some way to substituting a proxy arbiter of quality control.

With these points in mind, my aim here has been to present an overview of some of the key knowns and unknowns of SARS-CoV-2 adaptive immunity, relying both on preprints and on published findings. My focus has been to some extent informed by the recurrently posed questions that have clearly been of concern, whether in media interviews or policy discussions. Few in the immunology research community had prior, hands-on, experience with the immunology of coronaviruses, and one of the challenges has been to convey the notion that, as for any host defence programme, once you drill down beyond textbook generalities of viral immunity, the devil is in the detail, and that is what current research must resolve.

## THE VIRUS AND TARGET ANTIGENS

The spread of a fatal respiratory syndrome focused on initial cases who had visited Wuhan seafood market in China and was first reported in December 2019 [5, 6]. Within a little over 4 weeks of the initial case, genomic sequence for a novel coronavirus was published in early January 2020 [7]. It was initially termed 2019-nCoV and then, SARS-CoV-2. In terms of known human infections, it is phylogenetically close to SARS-CoV and is believed to be a new zoonotic transfer, probably from bats, although it is still unknown whether there was an intermediate species, such as pangolin [8]. Interestingly, a recently sequenced betacoronavirus from bats in Yunnan province, termed RmYN02, has 93% sequence identity with SARS-CoV-2, but with critical changes in receptor-binding domain (RBD) of the spike protein, meaning it probably lacks the key feature of binding to human angiotensin-converting enzyme 2 (ACE2) [9]. The coronaviruses have single-stranded RNA genomes encoding 16 non-structural proteins as well as structural proteins: spike, envelope, membrane and nucleocapsid. While host immunity may be revealed against any part of the viral proteome, much initial immunology research has focused on immunity to the spike antigen; this is driven by the knowledge that the interaction between the RBD within the spike antigen and human ACE2 is critical for viral entry and infectivity, and that antibodies against spike can be protective through neutralization. In this regard, many initial clues came from extrapolating from the immunology of the closely related infections caused by SARS-CoV and MERS [10–13].

## ANTIBODY TESTS AND FUNCTIONS

The genomic sequence was rapidly shared and published, facilitating design of PCR and antibody-based diagnostics [7]. An initial challenge in design of antibody tests, especially in the face of limited supply of positive control serum samples for testing, was to validate binding that would be specific to this virus [13]. The confounder of cross-reactivity with SARS or MERS antibodies is a concern, while prior exposure to the common cold HCoV viruses is considerably more widespread in human populations, though the genomic sequences show far lower conservation [14]. Most test strategies have relied on recombinant spike antigen. Some use the nucleoprotein, though this is more conserved across coronaviruses and therefore more prone to detect cross-reactive binding. While lab assays were validated and cohort antibody data accrued [10–14], there was a considerable public health imperative to scale-up antibody tests for patient screening and for seroprevalence studies [15]. Clearly, there is a range of approaches for antibody tests, covering lateral flow devices optimized to give a yes/no binary answer for antigen-binding, ELISA-based approaches, and then functional neutralization assays based either on pseudotype virus or on live virus assayed under BSL3 conditions. Rolling out reliable antibody testing at scale and at speed proved extremely challenging and has only recently been resolved, based largely on ELISA approaches.

By analogy to studies on SARS and MERS, most exposed, symptomatic individuals would be predicted to show an antibody response to spike antigen [16–19]. Furthermore, when tested for functional virus neutralization, this has tended to correlate well with total antigen-binding antibody by ELISA [12]. Data sets have now been shared for spike antibody in a large number of SARS-CoV-2 patient cohorts. A number of general points emerge from this: patients show a wide range of antibody titres, absence or near absence of antibody is associated with very mild infection, with fatal outcome or with immunodeficiency; the time course for appearance of IgM, IgG and IgA antibodies follows conventional kinetics over the initial 10–28 days of infection, the appearance of detectable antibody roughly contemporaneous with the disappearance of infectious virus from nasopharyngeal swabs; despite differential disease susceptibility between children, adults and the elderly, this is not reflected in simple quantitative differences of antibody titre [10–14, 19]. Some data indicate that cumulative viral load is correlated with antibody titre, raising concerns that those with very mild exposure may have more marginal or undetectable antibody responses, as was seen in SARS and MERS. However, while initial antibody studies were skewed to analysis of those ‘tip of the iceberg’ cases severe enough to be hospitalized, more data sets are now available for milder exposures such as healthcare workers, reassuringly showing significant antibody responses [20].

Chung and colleagues used a systems serology approach to investigate functional correlates of antibody responses across age groups and disease profile [21]. Systems serology harnesses the power of machine learning with data sets from multiple assays of antibody functionality including avidity and Fc receptor binding to generate correlative signatures [22]. They identified a convalescence biosignature associated with IgG3, FcγR binding and C1q engagement, as well as an influence of HLAII polymorphisms. There has been much speculation as to whether differential immune repertoires help to inform the differential susceptibility of the very young and the very old. This study offers the perhaps counterintuitive suggestion that children may benefit from an IgM-dominated signature, unlike the class-switched IgG and IgA signatures of the elderly. Differences across the lifespan may also relate to recent exposure to epitope-cross-reactive HCoV common cold viruses, though the functional extent of any such cross-reactivity and associated protection has been a source of controversy. A recent study by Ng *et al*. finds evidence for cross-reactivity between HCoV and SARS-CoV-2 spike and nucleoprotein, even to the extent of functional neutralization [14].

Since many key questions about durability of the antibody response and about correlates of protection have been hard to address in this short timeframe, there has been value in recourse to the coronavirus immunology literature, especially in relation to SARS and MERS [16–19]. This suggests a consensus of around 2 weeks to IgG seroconversion, lower responses in asymptomatic or mild infection, variably poor durability of antibody response beyond 1 or 2 years and neutralizing antibody as a likely correlate of protection. Certainly in follow-up of MERS patient, a significant minority showed no detectable antibody at 18 months.

More studies will be needed to ascertain whether the greater IgG repertoire of older individuals, including an HCoV cross-reactive repertoire, may contribute to enhanced pathogenesis through antibody-dependent enhancement [23].

Many teams have moved rapidly to express neutralizing human monoclonals characterize the binding site structural biology and evaluate potential translational use as therapeutics [24–26], following the rationale trialled in recent years for infections including HIV, Ebola and C. diff [27–29].

In some reported cohorts, there has been a minority of patients who have made a full recovery, seemingly without generating any detectable antibody response [30]. There are also reports of people unable to make any B-cell response at all due to agammaglobulinaemia who can also make a full recovery [31]. Whatever else, this suggests that neutralizing antibodies are not strictly required for recovery and other parts of the specific response, such as T cells, may serve to offer sufficient protection. The nuances of these emerging data sets have significant ramifications for clinical management of patients with inflammatory and autoimmune diseases across many specialties, weighing up the profile of their different disease-modifying therapy protocols to establish which are most likely to be safe.

Meanwhile, following many false starts, seroprevalence data sets are starting to appear from diverse urban locations around the globe. Some care is needed in collating these data as it can sometimes be hard to ascertain the precise sampling location and procedure, sample size, detection assay used or indeed whether derived from actual antibody tests or from predictive models. While results obviously look very different in different affected centres, seroprevalence levels seem typically to be in the 5–10% range, far short of the 60% plus needed for herd immunity, thus the urgent imperative for effective vaccines [32].

## T-CELL SUBSETS AND RESPONSES

Experience from T cell studies in SARS immune donors suggests that strong CD4 and CD8 immunity may endure for a number of years [32]. Initial analysis of immune subsets in acute COVID-19 largely focused on hospitalized patients with severe infection, though studies of the response during milder infection are now appearing [10, 33–38]. Analysis of the hospitalized cohorts shows a picture of large scale T-cell activation, especially CD8 cells, along with T cell lymphopaenia as a correlate of severity, and expression of exhaustion markers such as PD-1 and TIM-3 [34, 38].

CD4 and CD8 T-cell immune responses can be detected to diverse regions of the viral proteome including the nucleoprotein, spike/RBD and the main protease [35–37]. Antiviral T-cell immunity correlates with neutralizing antibody titres [10]. Peptide epitope mapping of antiviral T-cell responses has been taken to support a case for the possibility of cross-reactive protection by memory T cells recognizing epitopes shared with HCoV sequences [35]. Thieme *et al*. recently evaluated CD4 and CD8 responses against spike, membrane and nucleocapsid antigens, comparing between moderate, severe and critical disease. All three antigens contained epitopes, though the strongest response was to the membrane protein. The strongest responses, often polyfunctional, were seen in severe cases [36]. Should we be devoting extensive attention to the details of T-cell immunity if the data suggest that neutralizing antibody titre is itself a candidate correlate of protection? The answer is resoundingly in the affirmative, for several reasons. As we debate one of the biggest concerns around the fragile durability of coronavirus antibody responses, T-cell memory may be more enduring than B cell. Extrapolating from progress in influenza vaccinology, it is likely that the most efficacious vaccine approaches need to look to strong recognition by both B-cell and T-cell receptors [39]. Whether in the context of natural or vaccine-induced T-cell immunity, there is a need for some scrutiny of the elicited cytokine profile. For example, in SARS infection, Th2 cytokine responses can be a cause of lung immunopathology [40].

Another recent study reported a deep immune profiling analysis, comparing parameters in 71 patients compared to convalescent individuals or controls with respect to around 200 immune parameters and 30 clinical parameters [38]. The comprehensiveness of this analysis is useful for its ability to reinforce a number of observations suggested by smaller studies. The study reiterates the observations of others in relation to lymphopaenia, T-cell exhaustion, decreased Tregs and γδ T cells, and the notion of development of neutralizing antibody as a likely correlate of protection. Analysis of the parameters by tSNE-enabled delineation of distinct, clinically related, immune phenotypes including an activated immune profile associated with expanded B-cell plasmablasts and activated CD8 T cells that are positive for Ki67, HLA-DR, CD38, CD39, PD-1, ICOS and FAS.

Back to back papers from Barouch and colleagues using a macaque infection and challenge model show the benefits of relevant animal models for clarifying some of the unknowns, although, with the caveat that short-term rechallenge at 4 weeks is a poor proxy for understanding longer-term protection. The studies nevertheless show that either viral infection or DNA vaccination results in non-sterilizing protection from symptomatic disease and that neutralizing antibody may indeed be the best correlate of protection [41, 42].

## CYTOKINE STORMS AND THERAPEUTICS

From some of the earliest reports of serum biomarkers for severity of disease in patients who require ventilation, there has been evidence of a signature that includes inflammatory markers such as high ferritin, CRP, D-dimer and IL-6 [38]. Consistent reports of raised IL-6 led to a series of clinical trials with the therapeutic monoclonal, tocilizumab, in what appear to be promising trials [43]. Another recent study highlights a biosignature of CXCL10, CCL7 and IL-1 receptor antagonist associated with high viral load, impaired lung function, lung injury and lethal outcome [44]. We perhaps need some caution in overuse of the rather too simple term, ‘cytokine storm’, lest it blinds us to considering the aetiology and detail of what looks a rather unusual signature, worthy of investigation. Which cells have produced the response and to what specific viral stimulus? Certainly, this is not particularly reminiscent of a classic superantigen septic shock, to which it is sometimes related [45] and, for example, many classic T-cell-derived cytokines are not a feature here. Furthermore, there have been a number of reports of a Kawasaki-like disease in children, now numbered at several hundred cases [46], with a disease now provisionally termed, ‘Paediatric inflammatory multisystem syndrome temporally associated with SARS-CoV-2’ (PIMS-TS) [47]. Kawasaki disease itself still lacks a fully defined immunopathogenesis—it is considered an infection-triggered inflammatory cascade associated with the IL-1β pathway [48], and responding to therapeutics including IVIG.

## FUTURE DIRECTIONS

Despite rapid progress, there are rather urgent unknowns. This is an infection that affects different populations, whether in relation to age group or ethnicity, extremely differently, with old age by far the largest risk factor for severe outcome. While immunology has been able to offer hints as to the differential response that may underpin these differences, we still lack clarity. We are only starting to grapple with the detail of the diversity of cell types and tissues that can be infected by SARS-CoV-2. Understanding the nuances of infection, immunity and immunopathology in the lung, kidney and heart will be important. Note that ACE2 expression is relatively widespread across different cell-types, and we are only at the beginning of understanding the ramifications of this beyond the upper respiratory tract. While much has been learnt about immune correlates of infection, data so far are rather heavily weighted to analysis of severe, hospitalized patients. This leaves a vital knowledge gap to fill about the nature of immune recognition and immune memory of the many millions of individuals infected by the virus, either asymptomatically or symptomatically but managed in the home. Such individuals comprise the vast majority of the exposed population, and there is an urgent imperative to build the narrative as to the extent and features of their adaptive immune response. Perhaps the most critical aspect of this is the durability of that response, of concern with respect to mitigation of further waves. By analogy to the other human coronaviruses, the worrying prediction is that immunity may not commonly be durable beyond a few years.

Till now, all resources have necessarily been devoted to the firefighting of the response to the acute pandemic. Attention is gradually shifting to the need for a more granular understanding of the interplay between host immunity and damaging immunopathology, whether in lung disease, in the pathway leading to thrombosis, or at other sites. In the future, considerable healthcare resource will need to be allocated to COVID-19 follow-up clinics. The indications already are that, both among those who were severely affected and those that were not, there may a need to treat those living with long-term consequences of the infection, from lung fibrosis or bronchiectasis, to renal and cardiac complications and to chronic systemic, post-viral syndromes.

The real test of how far the fast track immunology pipelines have progressed will be whether effective vaccines can be widely delivered in the near future. While there has been much optimism about the likelihood or even, inevitability, of Phase I/II trials shifting promptly into wide-scale production and roll-out during 2020, the ups and downs of many other initiatives over the past decade caution against blithe assertions. There has at times been an unhelpful tendency to flip-flop between extremes of unrealistic optimism (‘definitely by September’) and unrealistic pessimism (‘we must accept then there may never be a vaccine’). As with any vaccine programme in history, there is a multitude of possibilities between these. Experience to date with SARS-CoV-2 suggests that this may not prove to be an infection that throws up insurmountable confounders to vaccine design—approaches that can safely and durably elicit neutralizing antibody look likely to work. The effort already has been unprecedented, spanning every known vaccinology platform, from adjuvanted conjugate vaccines, to attenuated or inactivated virus, to RNA and DNA vaccines and recombinant adenovirus approaches. While they are all well-established approaches with strong credentials and track records in a research setting, many have never before crossed the finish line to full manufacture and licensure. Each approach comes with its own nuanced strengths and weaknesses in terms of magnitude of response, durability of response, number of boosts likely to be required, ability to stimulate different aspects of mucosal or systemic immunity, bias in terms of B cells, T cells and T-cell subset polarization, safety profile, ease of manufacture and supply chain/storage issues. These are important questions that have thus far received little attention in the discussions framed as a simple race to the finish line. It will also be important to educate policymakers and the public about the nature and limits of clinical trials to avoid erosion of confidence through unrealistic expectations: it seems unlikely that any of the vaccines will elicit sterilizing or lifelong immunity, but ones that significantly mitigate the most severe disease manifestations would be ‘good enough’. On the contrary, under this intense scrutiny, any adopted candidates that are seen to be less good than this carry the risk of collateral damage for all other vaccines at a time of global health vulnerability under pressure from vaccine hesitancy.

This pandemic merits a response from the immunology community that promotes the ‘best answer’, which may not be the ‘first’. For this purpose, it is invaluable to foster coordinated endeavours through bodies such as CEPI, Gates Foundation and Wellcome, as an antidote to parochial ‘vaccine nationalism’ that risks the piecemeal development of rival, regional vaccines, which may impact both on control of infection and on equity of access. This is a global infection that crosses all borders—the specific language or nationality of the vaccine(s) is an irrelevance.
